# Plasma cell-free DNA as a sensitive biomarker for multi-cancer detection and immunotherapy outcomes prediction

**DOI:** 10.1007/s00432-023-05521-4

**Published:** 2024-01-09

**Authors:** Juqing Xu, Haiming Chen, Weifei Fan, Mantang Qiu, Jifeng Feng

**Affiliations:** 1 Department of Oncology, Jiangsu Cancer Hospital, Jiangsu Institute of Cancer Research, The Affiliated Cancer Hospital of Nanjing Medical University, Nanjing, 210009 China; 2 Department of Hematology and Oncology, Department of Geriatric Lung Cancer Laboratory, The Affiliated Geriatric Hospital of Nanjing Medical University, Nanjing, China; 3 Department of Thoracic Surgery, Peking University People’s Hospital, Beijing, China; 4 Thoracic Oncology Institute, Peking University People’s Hospital, Beijing, China

**Keywords:** Cell-free DNA, Multiple cancer, Whole-genome sequencing, Cancer detection

## Abstract

**Background:**

Cell-free DNA (cfDNA) has shown promise in detecting various cancers, but the diagnostic performance of cfDNA end motifs for multiple cancer types requires verification. This study aimed to assess the utility of cfDNA end motifs for multi-cancer detection.

**Methods:**

This study included 206 participants: 106 individuals with cancer, representing 20 cancer types, and 100 healthy individuals. The participants were divided into training and testing cohorts. All plasma cfDNA samples were profiled by whole-genome sequencing. A random forest model was constructed using cfDNA 4 bp-end-motif profiles to predict cancer in the training cohort, and its performance was evaluated in the testing cohort. Additionally, a separate random forest model was developed to predict immunotherapy responses.

**Results:**

In the training cohort, the model based on 4 bp-end-motif profiles achieved an AUC of 0.962 (95% CI 0.936–0.987). The AUC in the testing cohort was 0.983 (95% CI 0.960–1.000). The model also maintained excellent predictive ability in different tumor sub-cohorts, including lung cancer (AUC 0.918, 95% CI 0.862–0.974), gastrointestinal cancer (AUC 0.966, 95% CI 0.938–0.993), and other cancer cohort (AUC 0.859, 95% CI 0.776–0.942). Moreover, the model utilizing 4 bp-end-motif profiles exhibited sensitivity in identifying responders to immunotherapy (AUC 0.784, 95% CI 0.609–0.960).

**Conclusion:**

The model based on 4 bp-end-motif profiles demonstrates superior sensitivity in multi-cancer detection. Detection of 4 bp-end-motif profiles may serve as potential predictive biomarkers for cancer immunotherapy.

**Supplementary Information:**

The online version contains supplementary material available at 10.1007/s00432-023-05521-4.

## Introduction

Cancer is a leading cause of morbidity and mortality worldwide, and the number of newly diagnosed cancer cases per year is increasing (Sung et al. 2021). Effective diagnosis and prevention are key points for improving patient prognosis. Although new cancer screening methods are available, including low-dose computed tomography (LDCT) for lung cancer scanning and gastrointestinal endoscopy to detect gastroenteric tumors, the detection of tumors and survival rates remain unsatisfactory (Ajani et al. 2022; Benson et al. 2021; Blandin Knight et al. 2017). Their usage has been limited due to factors such as radiation exposure, invasion, high false-positive rates, and high costs. Therefore, there is an urgent need to develop a reliable, non-invasion, accurate, and cost-effective approach for detecting cancers.

Cell-free DNA (cfDNA) is a DNA fragment released into the bloodstream by cell apoptosis or necrosis (Kustanovich et al. 2019). In cancer patients, a fraction of cfDNA is released by tumor cells, which is termed circulating tumor DNA (ctDNA) (Aggarwal et al. 2021). Accumulating evidence suggests that circulating DNA fragments do not undergo a random fragmentation process. They carry genetic and epigenetic information from the cell and tissue of origin, which can be associated with tissue sources, disease status, chromatin accessibility, and nuclease activities (Chan et al. 2016; Jiang et al. 2018; Lo et al. 2021; Sun et al. 2015). As a result, cfDNA analysis, as a non-invasive approach, is increasingly finding applications in the fields of tumor diagnosis and treatment. The most prevalent method for cfDNA detection is somatic mutation sequencing. However, the sensitivity of mutation-based approaches may be compromised in patients with a limited number of recurrent mutations and the presence of non-tumor mutations resulting from clonal hematopoiesis of indeterminate potential (Cescon et al. 2020; Genovese et al. 2014). Additionally, several methylation-based cfDNA assays have been developed. Nevertheless, a significant challenge hindering its clinical implementation is the current incapability of existing detection technologies to simultaneously achieve high sensitivity, low cost, and deep sequencing coverage (Luo et al. 2021).

Recently, several cfDNA fragmentation-based approaches, including fragment size, end motifs, and nucleosome footprints, etc., have become new multi-omics technologies after ctDNA mutation and methylation (Garcia-Pardo et al. 2022; Wang et al. 2019). For example, tumor-secreted DNA tends to possess a shorter length compared to non-tumor-secreted DNA. Thus, detecting cfDNA fragment size can help distinguish cancer patients from healthy subjects (Chabon et al. 2020; Mouliere et al. 2018). However, the sensitivities of the approaches based on fragment size features may not be sufficient for clinical use in some tumors with low ctDNA shedding rates (Cristiano et al. 2019; Mouliere et al. 2018). Alternatively, analysis of cfDNA fragmentation features, including nucleosome position, occupancy and spacing (referred to as nucleosome footprints analysis) can reveal the tumor tissue-of-origin (Snyder et al. 2016; Vanderstichele et al. 2022). However, current approaches for cfDNA nucleosome remain challenging due to a lack of robust computational methods (Doebley et al. 2022). The profile of cfDNA end motifs represents a distinct type of plasma DNA fragmentation signature, revealing a large number of tumor derived changes (Jiang et al. 2020). As demonstrated by previous research, the hepatocellular carcinoma (HCC)-derived DNA fragments carry a different distribution of end motifs compared to non-tumoral DNA (Jiang et al. 2018). Moreover, a recent study revealed that patients with HCC exhibited a preferential pattern of cfDNA 4-mer end motifs compared to non-HCC subjects, with an AUC of 0.86 (Jiang et al. 2020). Such preferred end motifs were also observed among other cancer types (Jiang et al. 2020; Wang et al. 2023a). More importantly, the tumor-associated preferred end motifs are more pervasive, hence more readily detectable, and may therefore serve as an emergent class of ctDNA signatures. These findings collectively suggested that the end motif approach could outperform other fragment measures in identifying a variety of cancers (Cristiano et al. 2019). However, the utility of cfDNA end motifs in multiple cancer types still needs verification. Thus, we hypothesized that differences in cfDNA end motifs could enhance sensitivity for detecting cancer, as demonstrated by previous studies (Jiang et al. 2020, 2018) and facilitate non-invasive genomic analysis of cancer. In this study, we utilized 4 bp-end-motif profile to establish a robust model for detection multiple cancers. This model, based on 4 bp-end-motif, could also predict the response to immunotherapy. Our findings demonstrate that the proposed approach can aid in cancer detection and guide treatment.

## Methods

### Patient cohorts and sample collection

This study enrolled 106 individuals with histologically confirmed cancer and 100 healthy volunteers from the Affiliated Geriatric Hospital of Nanjing Medical University, China. The cancer cohort consisted of various cancer types, including lung cancer (33), gastric cancer (21), colon cancer (18), esophageal carcinoma (4), breast cancer (2), hepatocellular carcinoma (4), duodenal carcinoma (2), cholangiocarcinoma (2), renal cancer (4), bladder cancer (2), pancreatic cancer (3), ovarian cancer (3), endometrial cancer (1), mediastinal tumor (1), tongue cancer (1), thyroid cancer (1), cholangiocarcinoma (2), prostatic cancer, lymphoma (1), metastatic hepatic carcinoma (1), and lymph node metastasis carcinoma (1). Clinical characteristics, including immunotherapy information, were collected. The clinical information of individuals with cancer and volunteers without cancer is listed in Supplementary Table S2, 3. We performed plasma sample collection, shipping, and storage, cfDNA extraction, library preparation, and whole-genome sequencing (WGS) analysis uniformly as described in Supplementary Materials and Methods. In brief, the blood draw of the participants was performed from January 2022 to June 2022. The steps of cfDNA extraction, library preparation, and WGS were performed immediately after each other in batches by the College of American Pathologists (CAP)-accredited clinical laboratory (Beijing GenePlus Technology Inc., China). The study was approved by the ethics committee at the Affiliated Geriatric Hospital of Nanjing Medical University (approval no.014) and complied with the Declaration of Helsinki. All participants signed written informed consent forms.

### cfDNA extraction and whole-genome sequencing

We performed plasma sample collection, cfDNA extraction followed by WGS, as described in Supplementary Materials and Methods. Briefly, the venous blood samples were collected during routine physical checks (healthy volunteers) or on the day of therapy, prior to the first treatment (cancer patients). All samples were collected, shipped, and processed uniformly. A total of 5–10 ng of plasma cfDNA per sample was subject to PCR-free WGS library construction with the VAHTS^®^ Universal DNA Library Prep Kit for Illumina V3 (Vazyme). The libraries underwent paired-end sequencing on DNBSEQ-T7. To minimize bias, the sample operating team was blinded to the case or control status of the samples during the whole process.

### Bioinformatic analysis and modeling

Raw sequencing data processing was carried out as described in Supplementary Materials and Methods. The libraries in this study had a mean sequencing depth 5×. We extracted 4 bp-end-motif of cfDNA fragments from the WGS data for model construction. The cfDNA 4 bp-end-motif referred to the 5′ end 4 bp sequences, as reported by Jiang et al. (2020). The proportion and frequency of each 4 bp-end-motif over the total motifs (256, 4^4^) was calculated for each sample. Random forest (RF) models incorporating variable importance ranking were constructed and evaluated based on the training cohort using fivefold cross-validation as the resampling method to avoid overfitting of the model to new data. The predictive model’s performance was validated on the testing set.

### Statistical analysis

For statistical analysis, the receiver operating characteristic (ROC) curves were generated using the pROC package (1.18.4). Based on true positive (TP), true negative (TN), false positive (FP), and false negative (FN) of cancer prediction, we calculated the sensitivity [TP/(TP + FN)], specificity [TN/(TN + FP)], positive (PPV) [TP/(TP + FP)] and negative predictive values (NPV) [TN/(TN + FN)], accuracy [ (TP + TN)/(TP + FP + TN + FN)], as well as their corresponding 95% confidence intervals. Heatmap clustering analysis was generated using the pheatmap package (1.0.12) in R. The Mann–Whitney test, Fisher's exact test, and ANOVA analysis were performed using SPSS, and the Wilcoxon test was conducted using R.

## Results

### Participant characteristics

**Fig. 1:**
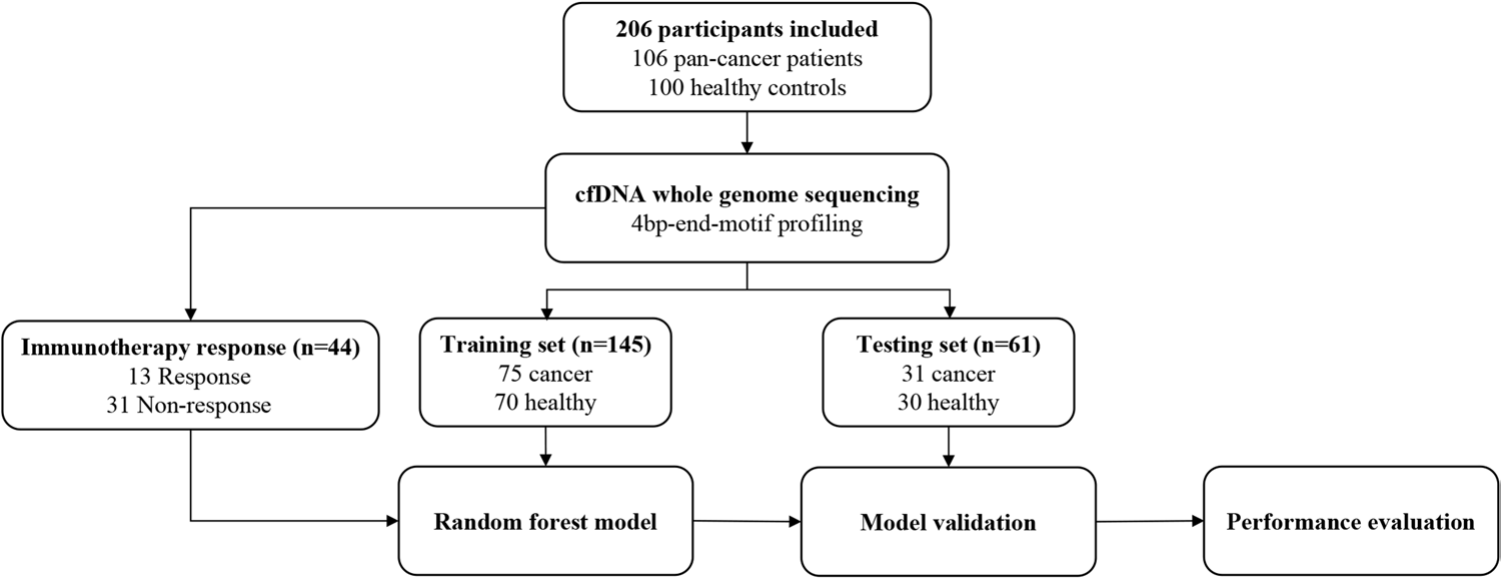
Workflow of the study. A total of 206 participants (cancer 106, healthy 100) were included in this study. Whole-genome sequencing of plasma cfDNA was performed, and their cfDNA 4bp-end-motif was profiled. 145 participants (cancer 75, healthy 70) were allocated to training for building the random forest algorithm-based machine learning model. 61 participants (cancer 31, healthy 30) were allocated to testing for confirming the model performance. 44 cancer patients administered immunotherapy (response 13, non-response 31) were allocated for building random forest algorithm-based machine learning model to predict the immune response

A total of 106 cancer patients treated in the Department of Hematology and Oncology, Affiliated Geriatric Hospital of Nanjing Medical University, were enrolled in the study. These patients represented 20 different cancer types, and their diagnoses and stages were assigned by treating physician according to the WHO classification. Among them, 2.8% were at stage II, 13.2% were at stage III, and 84.0% were at the stage IV. The cancer group had a median age of 67 (range 33–93), and the majority were male (60.4%) (Supplementary Table S2). Additionally, 100 healthy volunteers were collected from the Physical Examination Center, with a median age of 57 years (range 24–88), and males accounting for 27% of the participants (Supplementary Table S3). All participants were of Chinese origin. As shown in Fig. 1, the 206 participants were randomly assigned to the training cohort (75 multi-cancer and 70 healthy) and testing cohort (31 multi-cancer and 30 healthy) at a ratio of 7:3. The training cohort was employed for model construction and training, while the testing cohort was used for independent validation of selected feature variables and the corresponding model. The clinical characteristics of the participants are presented in Supplementary Table S1. Notably, there were uneven distributions in the mean age, sex, and BMI among patients with and without cancer within the cohorts. The proportion of females was higher in the healthy groups compared to the cancer groups (p_train_ < 0.001 and p_test_ = 0.012, Fisher's exact test), and the healthy volunteers were generally younger than the cancer patients (p_train_ = 0.002 and p_test_ = 0.243, Mann–Whitney test). As expected, the proportion of patients with BMI ≤ 24 was higher among cancer patients (p_train_ = 0.002 and p_test_ = 0.044, Fisher's exact test). Smoking status was comparable between the two groups. Despite these observed biases in the distribution of baseline clinical characteristics between cancer and non-cancer subjects, it’s important to note that these differences had no significant effect on the performance of the prediction model (P > 0.5, ANOVA analysis).

Of the cancer patients, 44 were administered immunotherapy, and 95.5% were at stage IV. The most common primary tumor observed were gastrointestinal (43.2%), lung (36.4%), and renal (9.1%) cancers. All patients received treatment involving the programmed death receptor 1 (PD-1) inhibitor, either alone or in combination with targeted agents, chemotherapy, or other immunotherapies. Among those who received immunotherapy, 13 patients (29.5%) achieving a response, while 31 patients (70.5%) displayed non-response to the treatment (Supplementary Table S2). A response was identified as the sum of complete response (CR) and partial response (PR), while non-response referred to patients who did not achieve CR or PR. CR was defined as disappearance of all target lesions. PR was defined as at least a 30% decrease in the sum of diameters of target lesions, taking as reference the baseline sum diameters (Eisenhauer et al. 2009).

### Model construction, validation, and statistical analysis

**Fig. 2:**
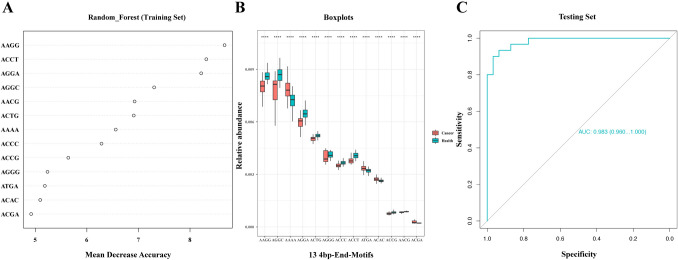
Predictive model construction and validation. **A** Variable importance plot from random forest analysis (mean decrease accuracy). The Mean Decrease Accuracy plot expresses how much accuracy the model losses by excluding each variable. The variables are presented from descending importance. The higher the value of mean decrease accuracy, the higher the importance of the variable in the model. **B** Boxplot showing differential frequencies between cancer and healthy subjects for the thirteen representative 4bp-end-motifs contributing most significantly to the model (****:* p* < 0.0001, Wilcoxon rank-sum test). X-axis represented the thirteen important variables from random forest analysis. Y-axis (relative abundance) represented the frequency of each motif which was calculated by dividing the number of reads carrying that motif by the total number of reads. **C** ROC curve evaluating the performance of predictive model in distinguishing cancer from healthy subjects for the testing set (AUC = 0.983)

Initially, we utilized the random forest algorithm to select important variables through variable importance ranking within the training set. We identified thirteen representative motifs contributing most significantly to the predictive model. Subsequently, we employed ROC curve analysis to study the potential diagnostic ability of the model for cancer detection. The AUC value between cancer patients and healthy participants was 0.962 with a sensitivity of 88.0% at 88.6% specificity (Supplementary Figure S1A, Table S4). Heatmap clustering analysis was then employed to discern the distinctive characteristics of these thirteen plasma motifs between cancer patients and healthy participants (Supplementary Figure S1B). This analysis revealed that the thirteen motifs tended to form distinct clusters between the two groups. Figure 2A also depicts the variable importance of features for the random forest model. AAGG was the most important of the thirteen factors, followed by ACCT and AGGA. Furthermore, we analyzed the frequency distribution of these thirteen selected variables across the 206 samples. The frequencies of all thirteen motifs showed significant differences between the cancer patients and healthy participants. Specifically, the frequencies of motif AAAA, ATGA, ACAC, and ACGA were significantly increased in cancer patients, while the frequencies of the remaining nine motifs (AAGG, AGGC, AGGA, ACTG, AGGG, ACCC, ACCT, ACCG, and AACG) showed a significant decrease in cancer patients (Fig. 2B). Previous studies have indicated that the sequence of end motif AAAA was highly expressed in hepatocellular carcinoma (HCC) samples and enriched in shorter sequences (< 150 bp) (Jiang et al. 2020; Jin et al. 2021). Consistent with prior findings, the abundance of end motif AAAA in our results was significantly increased in multi-cancer. However, data on the corresponding other sequences were not reported, which may be related to the different sequencing platforms. Despite this, considering that plasma cfDNA is contributed to the tissue of origin and that shorter fragments (< 150 bp) are predominantly derived from tumor secretion (Mouliere et al. 2018), our findings strongly suggested a preferential association of the end motifs AAAA, ATGA, ACAC, and ACGA with tumor-derived DNA ends. However, further research is needed to definitively establish if AAGG, AGGC, AGGA, ACTG, AGGG, ACCC, ACCT, ACCG or AACG are correlated with ctDNA fragment ends. Based on assessments in the testing cohort, the predictive model achieved an AUC of 0.962 and a sensitivity of 0.880 at a specificity of 0.886 (Fig. 2C, Table S4). These results suggest that our model exhibited an excellent ability to distinguish between cancer and healthy subjects.

### Performance of the predictive model in identifying multi-cancer

**Fig. 3:**
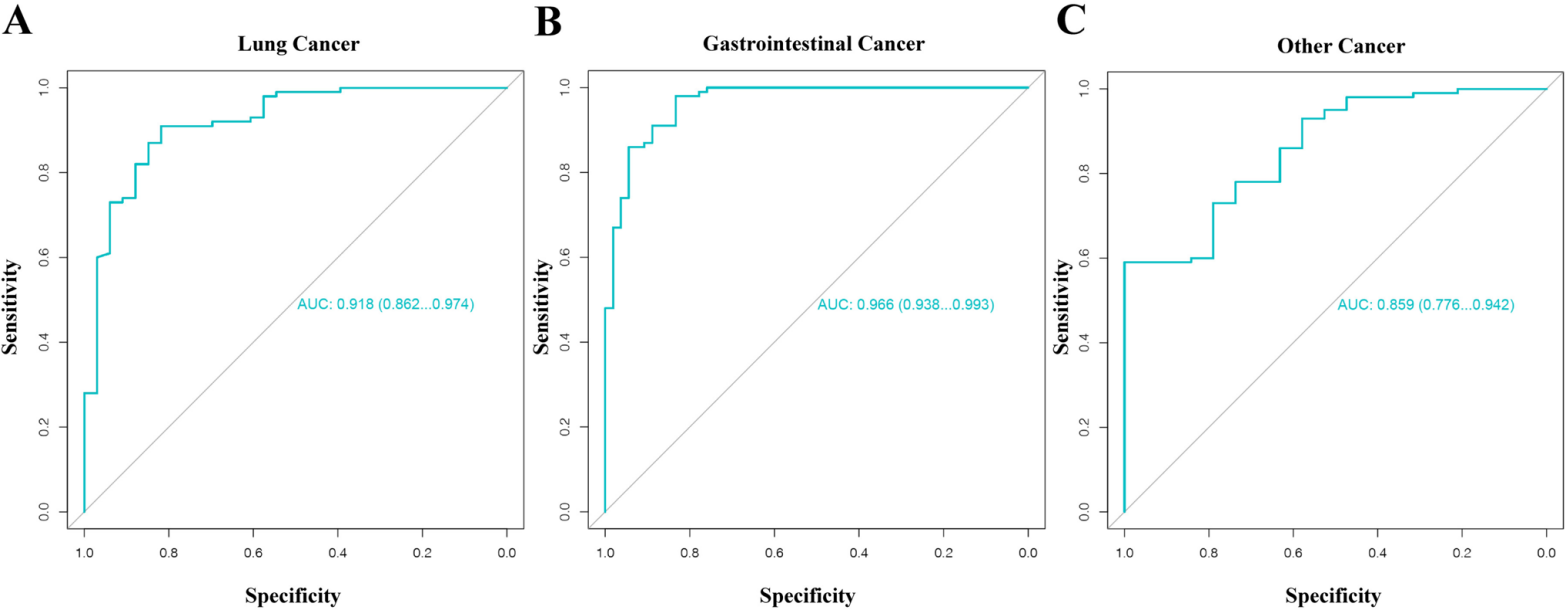
Evaluation the performance of predictive model in various cancer types. **A** ROC curve for the lung cancer cohort (AUC = 0.918). **B** ROC curve for the gastrointestinal cancer cohort (AUC = 0.966). **C** ROC curve for the other cancer cohort (AUC = 0.859)

**Fig. 4:**
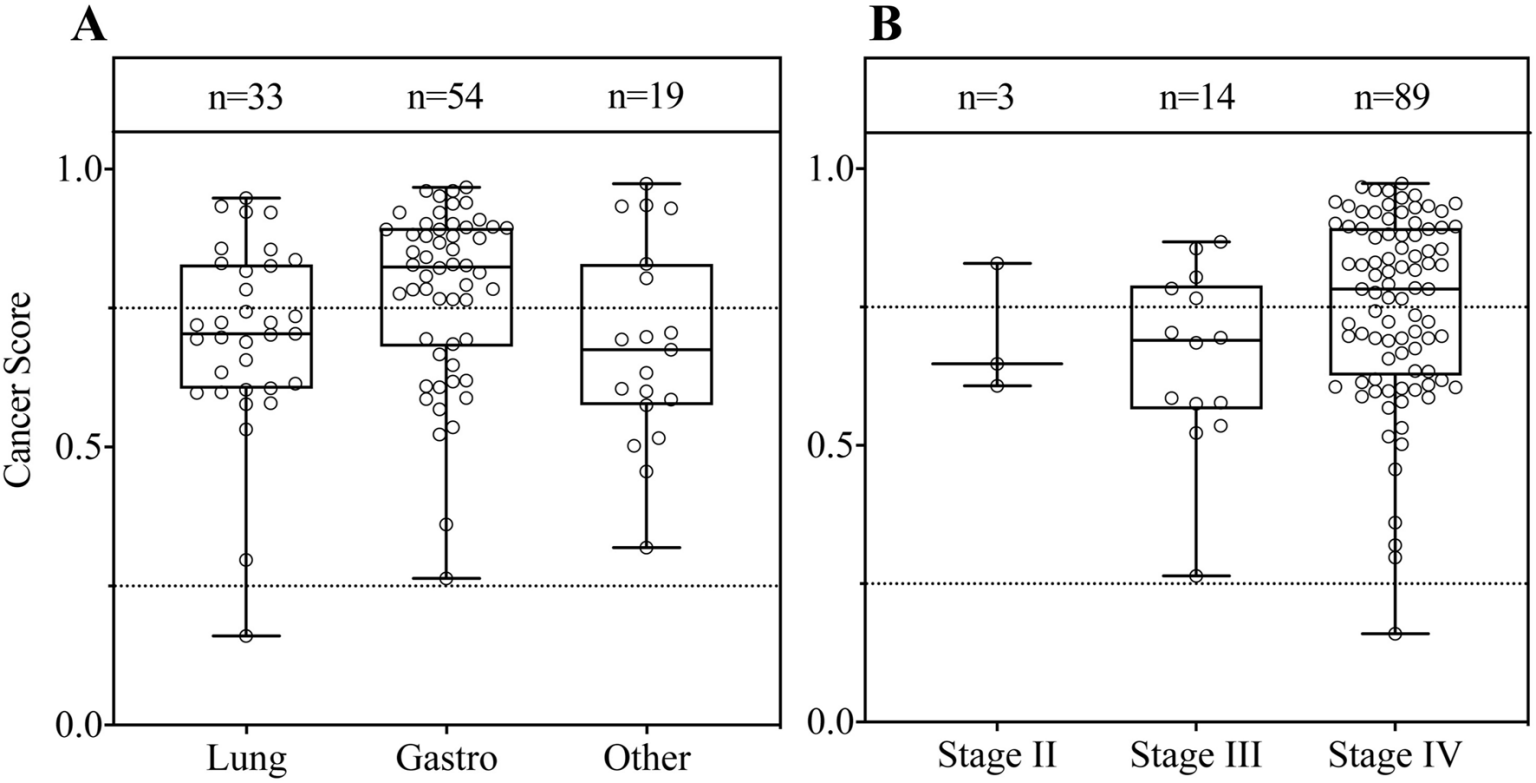
Distribution of cancer scores by cancer type (**A**) and cancer stage (**B**). Each dot in the boxplots represents the cancer score of each participant. The bar plot shows the mean value and standard deviation of each stage group. The case numbers in the groups are indicated

Subsequently, we examined the model’s performance across various types of cancer cohorts by combining the training cohort and testing cohort. A total of 106 multi-cancer patients were stratified into three sub-cohorts: lung cancer cohort (33), gastrointestinal cancer cohort (54), and the remaining types of tumors were grouped into the ‘other cancer’ cohort (19). The results consistently demonstrated that the model exhibited outstanding detection capabilities for each specific tumor type. As shown in Fig. 3 and Supplementary Table S5, the model’s AUC in the lung cancer cohort was 0.92 (95% CI 0.86–0.97), with sensitivity and specificity were 0.85 and 0.83, respectively. The model’s AUC in the gastrointestinal cancer cohort was 0.97, with a sensitivity of 0.94 at a specificity of 0.84. A similarly high level of performance was observed in the ‘other cancer’ cohort, achieving an AUC of 0.86 and a sensitivity of 0.63 at 0.82 specificity. Furthermore, we assessed the model’s performance across different cancer stages. For stage II–III, the AUC was 0.97 (95% CI 0.94–1.0) with a sensitivity of 1.0 and a specificity of 0.94. In stage IV, the model achieved an AUC of 0.96 (95% CI 0.94–0.97), along with a sensitivity of 0.97 and a specificity of 0.94. These results indicated that the model consistently delivered exceptional performance across various cancer stages. Additionally, it's worth noting that cancer scores exhibited variation not only across different cancer types but also across clinical stages, as visualized in Fig. 4.

### Prediction of tumor immunotherapy by cfDNA 4 bp-end-motif-based model

**Fig. 5:**
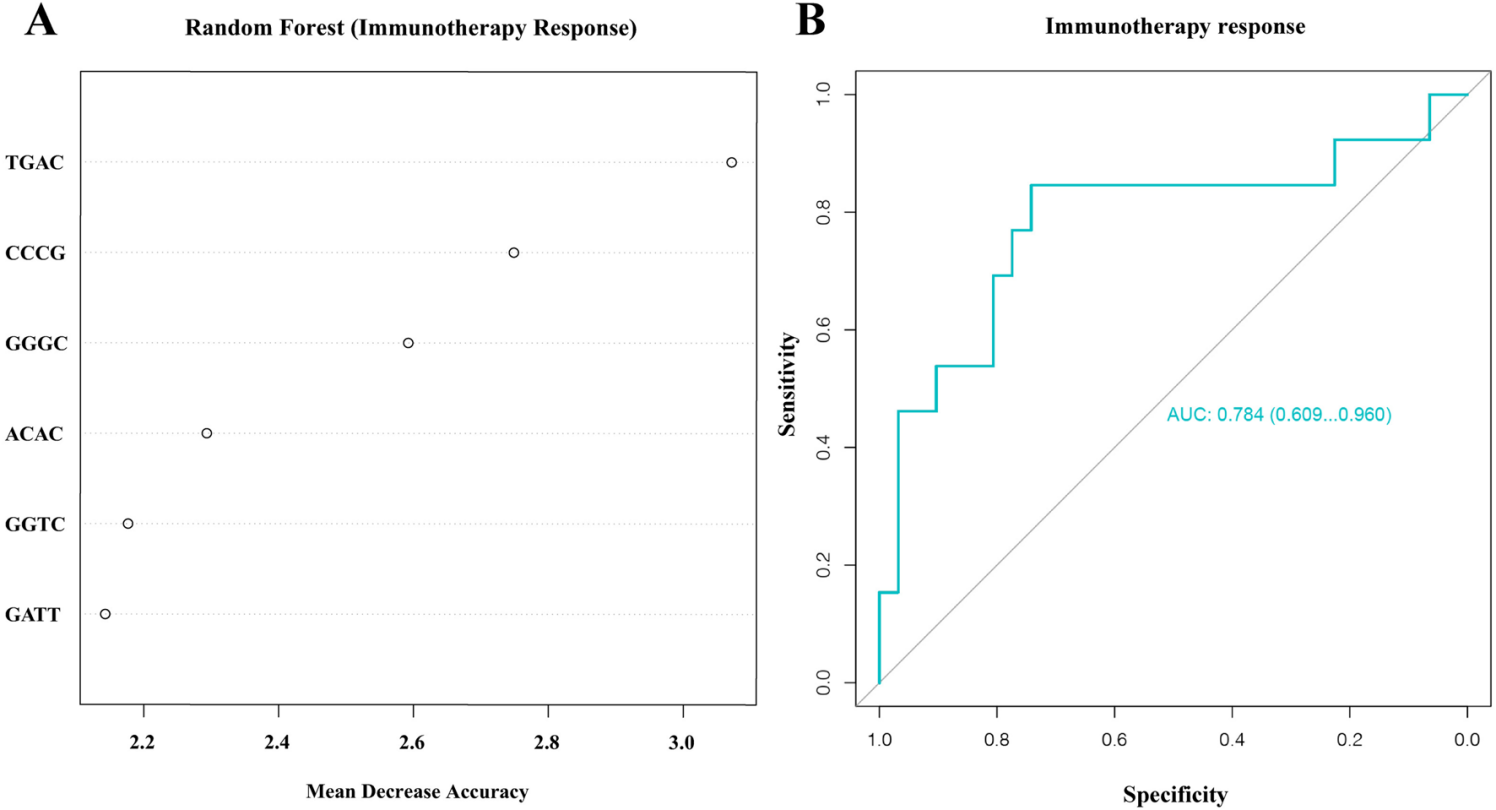
Immunotherapy prediction model construction and validation. **A** Random forest algorithm identifying six most representative motifs based on mean decrease accuracy. **B** ROC curve evaluating the performance of predictive model in distinguishing response from non-response for the patients receiving immunotherapy (AUC = 0.784)

To assess the value of cfDNA end motif in predicting response to immunotherapy across different tumor types, we evaluated the relationship between 4 bp-end-motif profiles and efficacy using immune check point inhibits. Among 44 immunotherapy-treated participants, 13 (29.5%) achieved a response, while 31 (70.5%) did not respond. We developed a random forest model and identified six most important variables (TGAC, CCCG, GGGC, ACAC, GGTC, and GATT) (Fig. 5A) through variable importance ranking. ROC curve analysis was employed to evaluate the potential predictive ability of immunotherapy outcomes. The AUC between response and non-response was 0.784 (95% CI 0.609–0.960) with a sensitivity of 0.846 and a specificity of 0.742 (Fig. 5B, Table S6). These findings suggest that pre-treatment plasma DNA end motifs hold promise for predicting immunotherapy outcomes.

## Discussion

In this report, we analyzed the 4 bp-end-motif feature derived from the WGS data, which identifies as the first 4-nucleotide sequence on each 5′ fragment end of plasma DNA after alignment to the reference genome (Jiang et al. 2020). Our findings demonstrated that profiling the distribution and sequences of cfDNA end motifs can effectively differentiate tumors from non-tumoral samples and can predict immunotherapy response.

In our study, the 4 bp-end-motif machine learning model, using the random forest algorithm, achieved an AUC of 0.983 (95% CI 0.96–1.00) and exhibited a sensitivity of 0.90 at a specificity of 0.87 in the testing cohort. In addition, the model showed excellent performance across various cancer stages, with high AUC values in both stage II-III (AUC: 0.97, 95% CI 0.94–1.0) and stage IV (AUC: 0.96, 95% CI 0.94–0.97). This performance surpassed that of other reported prediction models based on cfDNA end-motif profiles (Guo et al. 2022; Jiang et al. 2020; Wang et al. 2023a, b). Moreover, the model’s performance varied across different cancer types, with gastrointestinal cancer group showing the highest AUC (0.97, 95% CI 0.94–0.99), followed by lung cancer group (AUC: 0.92, 95% CI 0.86–0.97) and finally the other cancer group (AUC: 0.86, 95% CI 0.78–0.94). We noted an upward trend in the distribution of cancer scores from stage II to stage IV. Taken together these findings that the possible reason of our model outperform other existing models might be related to the association with late tumor stage and cancer-related heterogeneity (Bettegowda et al. 2014; Liu et al. 2020; van der Pol and Mouliere 2019). It’s worth noting that our model exhibited some biases in detecting different tumor types, likely due to an uneven sample proportion used in the model construction.

Previous study has suggested that the proportion of tumor-derived DNA fragment size is enriched in certain cancer types (e.g., liver, colorectal, lung, and breast) that shed more ctDNA into the bloodstream, while detection rates are lower in low-shedding cancer types like renal, bladder, pancreatic, and glioma (Mouliere et al. 2018). Notably, our study encompassed some cancers with ‘low-shedding rates’ classified within gastrointestinal cancer and other cancer types. Remarkably, the prediction model, constructed using 4 bp-end-motif, yielded high AUC values across various cancer types, indicating its consistent capacity to distinguish patients with cancer from non-cancer subjects. As mentioned earlier, cfDNA methylation-based methods have been used for multi-cancer detection, exhibited sensitivities for stage II–IV ranging from 0.43 to 0.93 (Liu et al. 2020). The sensitivity of our model was 1.0 in stage II–III, and 0.97 in stage IV for all cancer types, which is significantly better than their report. Furthermore, methylation-based methods usually rely on immunoprecipitation enrichment or targeted enrichment, limiting their ability to simultaneously achieve high sensitivity, low cost, and deep sequencing coverage (Luo et al. 2021). In contrast, our approach, focusing on plasma DNA end motifs, offers the advantage of achieving maximal diagnostic power with a relatively small number of DNA molecules analyzed (Jiang et al. 2020). These findings indicate that the detection of cfDNA end motifs is both highly sensitive and broad-spectrum, making it suitable for multiple cancer diagnosis (Jiang et al. 2020).

Recently, research has highlighted the role of cfDNA in predicting tumor recurrence and guiding treatment. For instance, a study by Y. Wang et al. revealed that cfDNA fragmentomics can potentially predict the response to neoadjuvant chemoradiotherapy (nCRT) in locally advanced rectal cancer (AUC: 0.96) (Wang et al. 2023b). Powles et al. reported that patients with urothelial carcinoma who tested positive for ctDNA were more sensitive to adjuvant atezolizumab (Powles et al. 2021). Additionally, our study reanalyzed 44 patients who received immunotherapy, and found that the baseline plasma 4 bp-end-motif based model exhibited high sensitivity in distinguishing responders from non-responders, achieving a sensitivity of 0.846 at a specificity of 0.742. These findings highlight the potential of utilizing pre-treatment cfDNA end motifs to identify patients likely to benefit from immunotherapy. Notably, our study is the first to demonstrate the potential of cfDNA end motif profiles as therapeutic biomarkers for immunotherapy in multi-cancer patients. However, the underlying mechanisms behind these observations remain poorly understood. Recent data have suggested that variations in plasma DNA profiles were associated with the deletion of the deoxyribonuclease 1-like 3 (DNASE1L3) gene. Serpas et al. showed that genetically inactivating the DNASE1L3 gene led to changes in the relative frequencies of cfDNA 4-mer end motifs (Serpas et al. 2019). Patients with DNASE1L3 gene deletion exhibited aberrations in size and a reduction of a ‘CC’ end motif of plasma DNA (Chan et al. 2020). Furthermore, many human cancers exhibit down-regulation of DNASE1L3 expression, and DNASE1L3 deficiency in mice led to delayed tissue recovery, increased chronic inflammation, immune cell dysfunction, impaired antitumor immunity, and ultimately affected the antitumor immune responses (Li et al. 2023; Liu et al. 2021). These findings suggest that the ability of end motif profiling to predict immunotherapy response may be linked to immune function dysregulation mediated by aberrant DNASE1L3 expression. Several biomarkers used to guide therapy selection, such as programmed death-ligand 1 (PD-L1) expression, tumor mutation burden (TMB), and microsatellite instability (MSI), often rely on invasive tumor tissue biopsies (Anagnostou et al. 2022). Additionally, minimal residual disease (MRD) has gained attention as a ctDNA-based detection approach primarily used for monitoring recurrence and guiding adjuvant immunotherapy in early-stage disease (Chaudhuri et al. 2017; Powles et al. 2021; Tie et al. 2022).However, there are limited reports on its role as a predictive marker for immunotherapy in advanced cancers. Collectively, our study demonstrates that cfDNA end motif profiles offer significant promise for non-invasive assessment and provide a new avenue for predicting clinical outcomes.

Several limitations exist in this study. Firstly, despite the remarkable performance in detecting multi-cancers and predicting immune response, the underlying mechanism of cfDNA end motifs remains not fully understood, necessitating further research into their mechanisms. Furthermore, the imbalance of baseline characteristics (age/gender/BMI) and the limited size of our sample population may introduce bias. Expanding the sample size and equilibrating the demographics in future studies will enhance the statistical power and provide more accurate estimates of the model’s predictive performance. Additionally, our study lacks a benign lesion group of comparison, which means our results do not offer insights into the model’s ability to discriminate between cancer and benign lesions. Further validation with independent and large-scale cohorts is necessary to address this limitation.

In conclusion, our study supports the nation that detecting the 4 bp-end-motif feature holds promising clinical application for cancer detection and provides profound insights for the design of personalized treatment strategies.

### Supplementary Information

Below is the link to the electronic supplementary material.

Evaluation of the predictive model based on 4bp-end-motifs in training set. (A) ROC curve evaluating the performance of the predictive model in distinguishing cancer from healthy subjects for the training set (AUC = 0.962). (B) The heatmap clustering analysis assessing frequencies of the thirteen end motif between cancer and healthy subjects (TIF 21351 KB)

Supplementary file2 (DOCX 22 KB)

Supplementary file3 (XLSX 749 KB)

## Data Availability

The raw data that support the findings of this study are available from the corresponding author upon reasonable request.
