# Supplementary figures and images for: Plasma cell-free DNA as a sensitive biomarker for multi-cancer detection and immunotherapy outcomes prediction
Source: J Cancer Res Clin Oncol. 2024 Jan 9;150(1):7. doi: 10.1007/s00432-023-05521-4 (PMC10776501; doi:10.1007/s00432-023-05521-4)

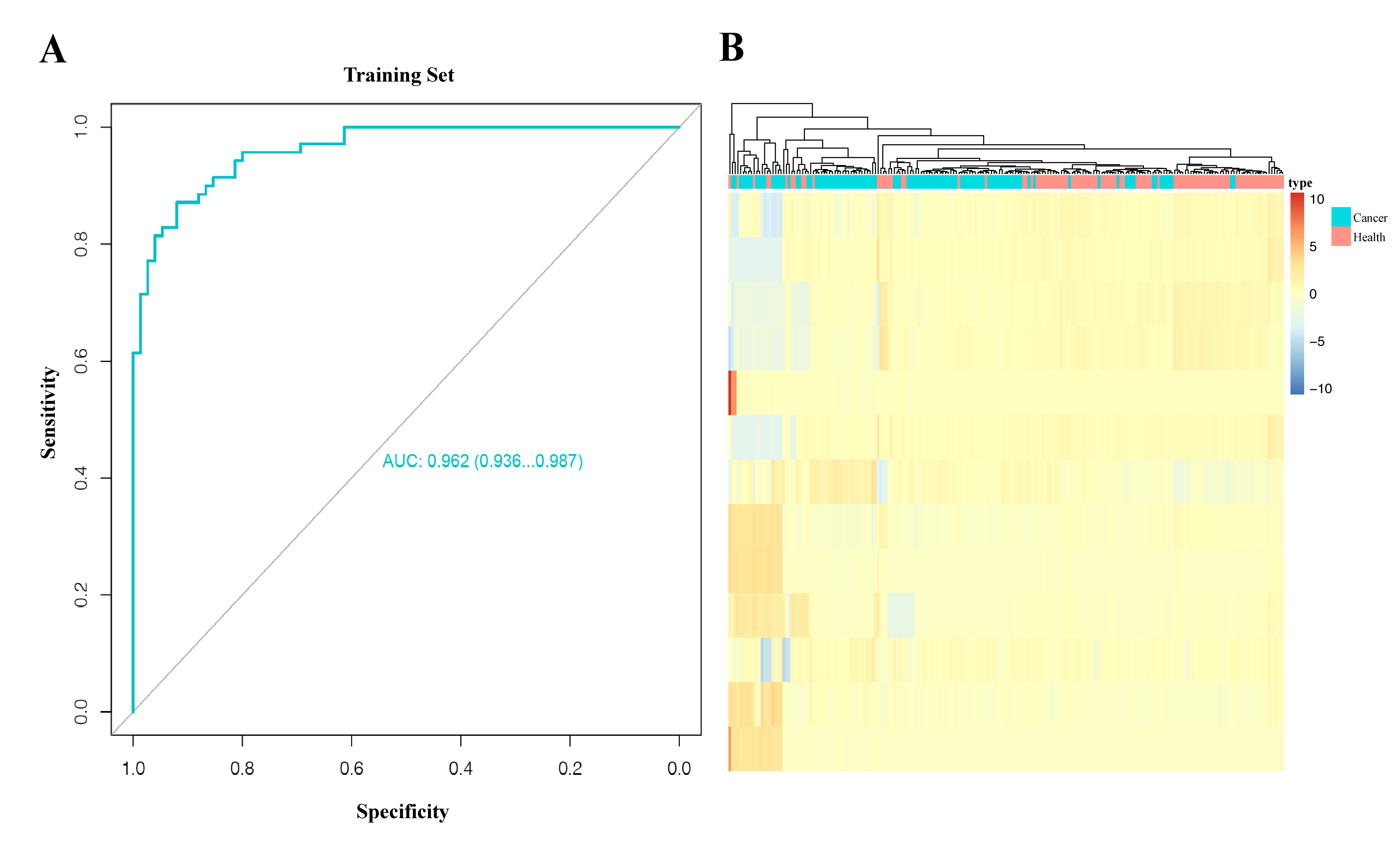

Supplement: Supplementary file 1 — Evaluation of the predictive model based on 4bp-end-motifs in training set. (A) ROC curve evaluating the performance of the predictive model in distinguishing cancer from healthy subjects for the training set (AUC = 0.962). (B) The heatmap clustering analysis assessing frequencies of the thirteen end motif between cancer and healthy subjects (TIF 21351 KB) [file 432_2023_5521_MOESM1_ESM.tif]
