# Supplementary material for: Plasma cell-free DNA as a sensitive biomarker for multi-cancer detection and immunotherapy outcomes prediction
Source: J Cancer Res Clin Oncol. 2024 Jan 9;150(1):7. doi: 10.1007/s00432-023-05521-4 (PMC10776501; doi:10.1007/s00432-023-05521-4)
Supplement: Supplementary file 2 — Supplementary file2 (DOCX 22 KB) [file 432_2023_5521_MOESM2_ESM.docx]

**Supplementary Materials and Methods**

**Participant enrollment**

A total of 106 patients with cancer and 100 healthy volunteers from the Affiliated Geriatric Hospital of Nanjing Medical University were enrolled in the study. The inclusion criteria for the patients include 1) age 18 years or older; 2) able to provide sufficient and qualified blood samples; 3) able to provide the written informed consent; 4) had complete medical record; 5) pathological diagnosis of cancer. The exclusion criteria include 1) currently diagnosed with other types of tumors; 2) participants during pregnancy or lactation; 3) any other disease/condition deemed not suitable for study enrollment by researcher.

The inclusion criteria for healthy volunteers include 1) age 18 years or older; 2) able to provide sufficient and qualified blood samples; 3) able to provide the written informed consent; 4) had no history of cancer diagnosis/treatment. The exclusion criteria include 1) participants during pregnancy or lactation; 2) participants of ongoing fever or recipient of anti-inflammation therapy within 14 days prior to study blood draw.

**Extraction of cfDNA fragment profile**

Blood samples collected from participants underwent cell-free DNA (cfDNA) extraction followed by whole-genome sequencing (WGS). We performed sample collection, cfDNA extraction, and WGS uniformly as follows: ~10 mL venous blood samples were collected during routine physical checks (healthy volunteers) or pre-treatment (cancer patients) into EDTA blood collection tubes (Becton Dickinson). Samples were kept at 4℃ for no more than 2 hours prior to centrifugation. Blood samples were centrifuged at 1,800 g for 10 mins at 4℃ for plasma collection. Plasma samples were centrifuged a second time at 16,000 g for 10 mins at 4℃ to remove cell debris. Then samples were frozen, shipped to the College of American Pathologists (CAP)-accredited clinical laboratory (Beijing GenePlus Technology Inc., China) on dry ice, and stored at -80℃ to for processing. The extraction of 10 to 250ng of cfDNA was performed automatically on Hamilton Microlab STAR automated liquid handling platform (Hamilton Company). The concentration was measured by Qubit dsDNA HS Assay Kit (Thermo Fisher Scientific). 5-10 ng of cfDNA per sample was subject to PCR-free WGS library construction using the VAHTS® Universal DNA Library Prep Kit for Illumina V3 (Vazyme), quantified using the KAPA Library Quantification kit (Roche), and underwent 100-bp paired-end sequencing on DNBSEQ-T7. Of note, the cfDNA extraction, genomic library preparation, and sequencing steps were conducted immediately after each other by the central lab following their standard operating procedures.

For bioinformatic analysis, FastQC was used for raw sequencing data quality control (QC) protocol^1^. After aligning the sequencing reads to the human reference genome hg19, we obtained the features of cfDNA motif profiles^2^. These motif features can classify samples based on the sequences at the end of cfDNA fragments. This study selected 4bp-end-motif as the research subject. The 4bp-end-motif feature referred to the 5’ end 4bp sequences, which was initially reported by Jiang et al^3^. In 256 possible 4bp-end-motifs, the frequency of each motif was calculated by dividing the number of reads carrying that motif by the total number of reads, generating an end motif feature vector of a length of 256 for each sample.

**Model Construction and validation**

The data samples, comprising 106 patients and 100 volunteers, were randomly divided into a training cohort (75 multi-tumor and 70 healthy) and a testing cohort (31 multi-tumor and 30 healthy) following a ratio of 7:3. This allocation was performed using the R “caret” package and “caTools” package (Supplementary Table 1 and 2). The random forest (RF), a machine learning approach, was developed on the training set and independently validated on the testing set using the “randomForestSRC” (2.12.1) package. The RF algorithm was employed to identify important variables through variable importance ranking and construct the model. The RF algorithm estimates the importance of a variable by using the mean decrease in accuracy upon removal of the variable. The more the accuracy suffers, the more important the variable is for the successful classification. The variables are presented from descending importance. The higher the value of mean decrease accuracy, the higher the importance of variable in the model. The model generated a cancer score for each sample representing the cancer possibility. To avoid overfitting of the model to new data, 5-fold cross-validation was utilized as the resampling method. The characteristics of the final classifier with the largest area under the ROC curve were estimated on the training set with 100-time 5-fold cross-validation. The heatmap clustering analysis was generated using the pheatmap package (1.0.12) in R. Boxplot analysis was used to evaluate the frequency distribution of thirteen selected variables in 206 samples. Ultimately, the final classifier was trained on the full training set and the performance was estimated on the testing set.

**References**

1. Bolger AM, Lohse M, Usadel B: Trimmomatic: a flexible trimmer for Illumina sequence data. Bioinformatics 2014, 30:2114-2120.

2. Li H, Durbin R: Fast and accurate short read alignment with Burrows-Wheeler transform. Bioinformatics 2009, 25:1754-1760.

3. Jiang P, Sun K, Peng W, et al. Plasma DNA End-Motif Profiling as a Fragmentomic Marker in Cancer, Pregnancy, and Transplantation. Cancer Discov 2020; 10(5): 664-73.
